# Delivering equitable care for patients with disabilities

**DOI:** 10.1016/j.jdin.2022.10.001

**Published:** 2022-10-28

**Authors:** Margaret A. Kaszycki, Grazyna Kaszycki, William Notaro

**Affiliations:** a Quinnipiac Frank H. Netter MD School of Medicine, North Haven, Connecticut; b Internal Medicine Department, Southbury Training School, Southbury, Connecticut; c Dermatology Department, Nuvance Health, Danbury, Connecticut

**Keywords:** economic, intellectual disabilities, health services, management, medical dermatology

An estimated 1% of the general population is living with an intellectual disability (ID), with 0.6% living with severe ID.^1^ The prevalence of skin conditions among the population with ID is 10% to14% greater compared with the general population; therefore, access to high-quality dermatologic care is clear.^2^ Unfortunately, most medical students and residents do not receive formal training regarding unique adaptations required when caring for patients with ID. Some students even reported developing negative perceptions toward patients with disabilities.^3^ Compounded by the socioeconomic inequalities and high demand for dermatologists, high-quality dermatologic care is not always achieved for patients with disabilites.^4^ Patient immobility, poor eyesight, behavioral and emotional lability, and polypharmacy all pose as diagnostic and management challenges. When a dermatologic consult is required, patient transport, behavior, triggers, and communication all need to be considered. Therefore, to address the quality of care, management, and economic issues in our group home for the intellectually disabled, we provided dermatologic care through a regularly scheduled visiting dermatologist.

Previous articles have highlighted the benefits of dermatologic visits to nursing homes 3 to 4 times a year,^5^ and we similarly report that dermatologic visits 4 to 5 times a year have also proven to be effective in a group home setting. We found many patient triggers can be avoided this way as patients need not travel to unfamiliar places and can be cared for and comforted in their familiar environments. Group homes are routinely equipped with basic medical equipment; therefore, creating an agreement regarding covering costs and storing of dermatologic equipment was not an issue. Additionally, as the nursing staff has become more familiar with our visiting dermatologist, they have been able to assist with biopsies and are familiar with postcare instructions for biopsies and cryotherapy treatments.

There is also a socioeconomic benefit as each patient does not need to be transported to the outside office individually, thus reducing operating costs and staffing issues. These issues have especially been prevalent during the COVID-19 pandemic and the rising costs that our economy is currently facing. Our facility and patients are very fortunate to have an experienced dermatologist visiting bimonthly who spends 90 to 120 minutes seeing a total of 8 to 10 patients previously identified by the nursing facility among a population of approximately 75 patients (Fig 1). Although we acknowledge that there is a shortage of dermatologists, we would like to emphasize on brevity, infrequency of visits, and schedule flexibility. The most frequent conditions treated by our visiting dermatologist include atopic dermatitis, factitious dermatitis, irritant dermatitis, intertrigo, and identification of precancerous or cancerous lesions. Additionally, the group home physician may be present and a shared decision can be made more effectively without any delays in initiating treatment. This allows for improved quality of care and patient management.

**Fig 1:**
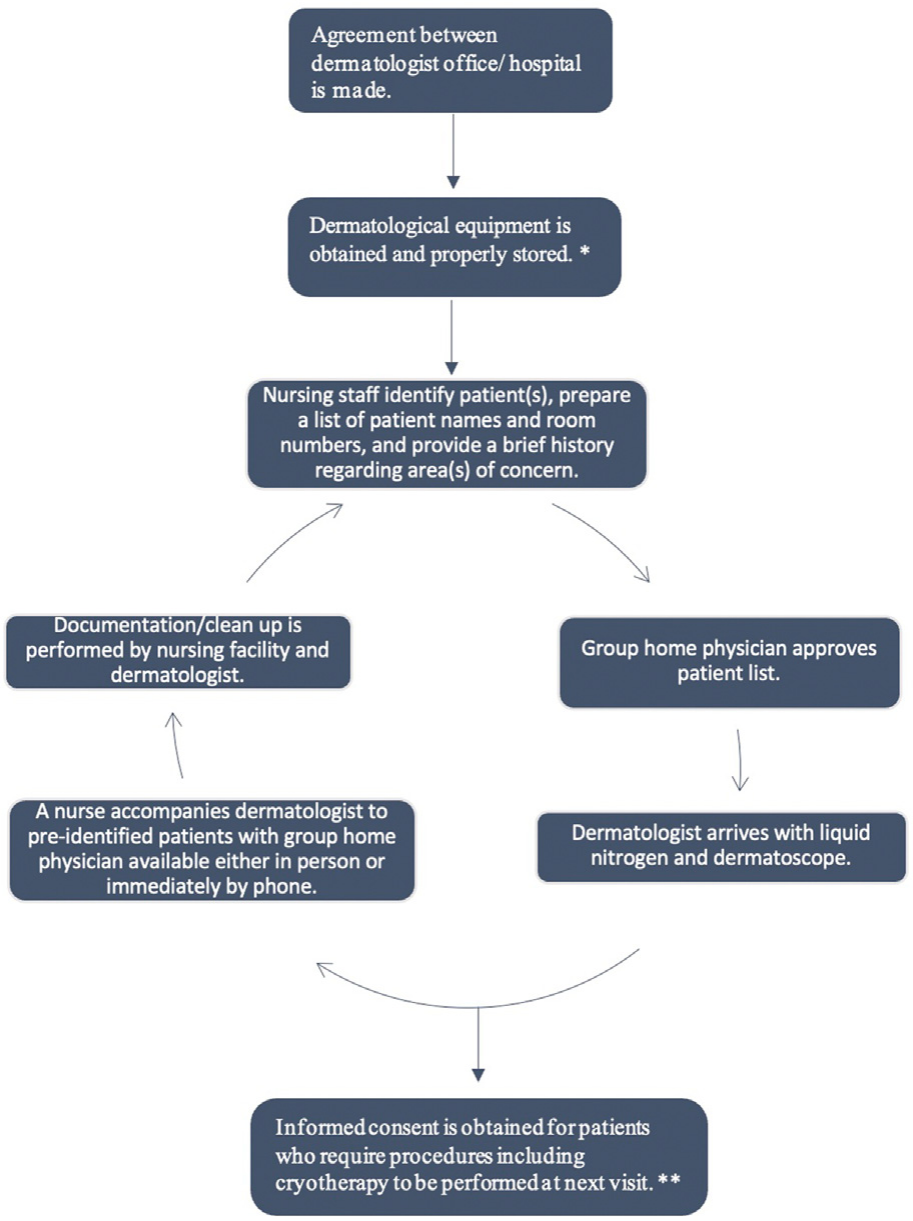
Workflow of the visiting dermatologist. ∗ Dermatologic equipment includes handles, scalpel and double edged razor blades, dermal punches, scissors, forceps, suture kits, gauze pads, specimen containers, aluminum chloride, petroleum jelly, and band aids. ∗∗ Informed consent obtained on the same day for urgent procedures.

Lastly, we would like to urge teaching facilities to create more inclusive patient populations in clinical scenarios to break down the stigma and negative perceptions toward patients with ID. After all, it is our duty and privilege to provide equitable care for all our patients.

## Conflicts of interest

None disclosed.

## References

[1] Maulik P.K., Mascarenhas M.N., Mathers C.D., Dua T., Saxena S. (2011). Prevalence of intellectual disability: a meta-analysis of population-based studies. Res Dev Dis.

[2] Jansen D.E., Krol B., Groothoff J.W., Post D. (2004). People with intellectual disability and their health problems: a review of comparative studies. J Intellect Disabil Res.

[3] Ankam N.S., Bosques G., Sauter C. (2019). Competency-based curriculum development to meet the needs of people with disabilities: a call to action. Acad Med.

[4] Peacock G., Iezzoni L.I., Harkin T.R. (2015). Health care for Americans with disabilities—25 years after the ADA. N Engl J Med.

[5] Klapwijk M.S., Kouwenhoven S.T.P., Achterberg W.P., Vermeer M.H. (2019). Dermatological consultations in a nursing home. Jour Nursing Home Res.

